# Interaction between microbiota and immunity and its implication in colorectal cancer

**DOI:** 10.3389/fimmu.2022.963819

**Published:** 2022-07-29

**Authors:** Changsheng Xing, Yang Du, Tianhao Duan, Kelly Nim, Junjun Chu, Helen Y. Wang, Rong-Fu Wang

**Affiliations:** ^1^ Department of Medicine, Keck School of Medicine, University of Southern California, Los Angeles, CA, United States; ^2^ Norris Comprehensive Cancer Center, Keck School of Medicine, University of Southern California, Los Angeles, CA, United States; ^3^ Department of Pediatrics, Children’s Hospital Los Angeles, Keck School of Medicine, University of Southern California, Los Angeles, CA, United States

**Keywords:** colorectal cancer, microbiota, innate immunity, adaptive immunity, colitis, metabolites, immune signaling, immunotherapy

## Abstract

Colorectal cancer (CRC) is one of the leading causes of cancer-related death in the world. Besides genetic causes, colonic inflammation is one of the major risk factors for CRC development, which is synergistically regulated by multiple components, including innate and adaptive immune cells, cytokine signaling, and microbiota. The complex interaction between CRC and the gut microbiome has emerged as an important area of current CRC research. Metagenomic profiling has identified a number of prominent CRC-associated bacteria that are enriched in CRC patients, linking the microbiota composition to colitis and cancer development. Some microbiota species have been reported to promote colitis and CRC development in preclinical models, while a few others are identified as immune modulators to induce potent protective immunity against colitis and CRC. Mechanistically, microbiota regulates the activation of different immune cell populations, inflammation, and CRC *via* crosstalk between innate and adaptive immune signaling pathways, including nuclear factor kappa B (NF-κB), type I interferon, and inflammasome. In this review, we provide an overview of the potential interactions between gut microbiota and host immunity and how their crosstalk could synergistically regulate inflammation and CRC, thus highlighting the potential roles and mechanisms of gut microbiota in the development of microbiota-based therapies to prevent or alleviate colitis and CRC.

## 1 Introduction

Colorectal cancer (CRC) is one of the most common cancers and a major health burden in the world, which accounts for about 10% of all new cancer cases globally and becomes the second leading cause of cancer-related death (1–3). About half of the human population will develop at least one benign colonic adenomatous polyp during their lifetime, with ~3% of these cases developing into CRC (4). Besides genetic alterations, colonic inflammation is a major environmental risk factor for CRC development, as the individuals diagnosed with ulcerative colitis (UC) and Crohn’s disease (CD), the most common types of inflammatory bowel diseases (IBDs), have markedly increased risk of developing CRC (5–7). Although immune cells, cytokines, and microbiota components contribute to colitis and CRC in a context-dependent manner (8, 9), the precise mechanisms remain largely unclear.

The human gut is a complex ecosystem composed of 10^13^-10^14^ bacteria, and these microbiota components play critical roles in controlling digestion and benefiting many aspects of human health. Some probiotics have been exploited as food supplements to support the health of the immune and digestive system, and even as novel therapies for disease treatment. Numerous studies have demonstrated that the microbiota composition is significantly altered in both IBD and CRC patients, compared with healthy people. Recent metagenomic profilings have identified multiple prominent CRC-associated bacteria enriched in patients, including the components in genera *Fusobacterium*, *Peptostreptococcus*, *Porphyromonas*, *Prevotella*, *Parvimonas*, *Bacteroides*, and *Gemella* (10). As most studies link mixed microbiota composition to disease progression, some species have been reported to promote colitis and CRC (11–14); however, only a few bacterial species or strains are identified as immune modulators that induce potent protective immunity. In this review, we provide an overview of the crosstalk between gut microbiota and host immunity and how it regulates inflammation and CRC development. Importantly, microbiota has been implicated in cancer immunotherapy. Due to the lack of clinical evidence on the functions of viruses and fungi, we will mainly focus on bacteria species, as the major component of microbiota.

## 2 Genetic risk factors in CRC development

CRC is a heterogeneous disease associated with a number of genetic mutations (15), with 10%-20% of all patients possessing a positive family history (16). Besides traditional methods, genome-wide sequencing analyses have been performed to depict the genomic landscape and transcriptome profile, thus allowing the establishment of key promoting and suppressing alterations in CRC development (17).

### 2.1 Genetic alterations in epithelial cells

Most CRC cases arise from an aberrant crypt on the colonic epithelium, which gradually develops into an adenoma, and ultimately adenocarcinoma (18). The genetic alterations in colon epithelial cells, as the primary site for CRC development, play critical roles in the disease transformation (19). To date, several hundred driver genes have been identified to promote neoplastic transformation by intragenic mutations, with other mutations being passengers that are not associated with selective growth advantages (20). It has been widely accepted that mutations in three major gene clusters, APC, KRAS, and TP53, are sufficient to initiate CRC (21, 22), while BRAF, PIK3CA, and SMAD4, which are also listed as the most frequently mutated genes in CRC (23), are identified as important drivers to promote the progression (24).

Although similar sets of oncogenes are involved, the genetic alterations between sporadic and colitis-associated CRC (CAC) are different in timing and frequency. In sporadic CRC, loss of APC function is a key event to initiate an adenoma, followed by the activation of KRAS, COX-2, and other factors; whereas the abnormality of TP53 usually occurs in the late stage of disease progression and drug resistance (25, 26). On the contrary, in CAC that arises from flat dysplastic mucosa, TP53 mutation is frequently detected in inflamed tissues and is an important step in early cancer development. APC mutation and Wnt dysfunction are relatively infrequently in CAC and occur in the late stage (25, 26).

Interestingly, the pathogenesis and molecular characteristics of CRC also depend on the anatomical locations of the tumors (27), particularly between the proximal (right-sided) and distal (left-sided) colons. Right-sided colon cancer is characterized by the alterations in BRAF, KRAS, and PIK3CA, and has a higher rate of deficient mismatch repair; whereas instability pathway-related APC and TP53 mutations are more frequently observed in left-sided colon cancer, which has a better response to both chemotherapies and targeted therapies, thus showing better prognosis in patients (28, 29). Meanwhile, rectal cancer is featured by the mutations in all three major genes (APC, KRAS, and TP53) and HER2 amplification, and has a lower rate of deficient mismatch repair (29).

### 2.2 Immune cell-related genetic alterations in CRC development

Besides alterations in colon epithelia as the primary foci, aberrant changes in the immune microenvironment also have profound impacts on the initiation and progression of CRC. One major evidence is that the risk of CRC development is increased in IBD (inflammatory bowel disease) patients (5, 6). It has been demonstrated that the age at diagnosis of IBD-associated CRC is 15-20 years earlier compared to sporadic cancers (30, 31), and CRC accounts for approximately 10%-15% of all deaths in IBD patients (32).

Given the critical roles of the host immune cells and cytokines in controlling CRC development, genetic alterations on immune-related genes or in immune cells, in addition to colon epithelial cells, are equally important for CRC pathogenesis. For instance, IL-10 is an immunoregulatory cytokine that plays a central role in controlling intestinal inflammation (33). Mice deficient in either IL-10 or its receptor develop spontaneous colitis, and became one of the most widely used animal models for studying IBD pathogenesis (34, 35). Furthermore, deficiency in ubiquitin ligase *Itch* leads to spontaneous colitis and increased susceptibility to CRC through the release of RORγt degradation and excessive production of IL-17A (36). A study shows that the deficiency of *Fam64a* in mice decreases Th17 cells and ameliorates colitis and CRC (37). These studies indicate that modulation of Th17 cell-related genes has a significant impact on CRC development, although the pro-inflammatory or suppressive roles of these Th17 cells are not validated in detail.

We recently showed that the myeloid-specific deletion of *Tak1* (*Tak1^flox/flox^;Lyz2-Cre^+/+^*) renders complete resistance of mice to DSS-induced acute colitis and AOM/DSS-induced CRC (38). Notably, gut microbiota compositions are completely altered in *Tak1^flox/flox^;Lyz2-Cre^+/+^* mice, compared to wild-type mice. Among them, *Odoribacter splanchnicus* is markedly accumulated and synergistically cooperated with IL-1β/IL-6 signaling pathways to induce and expand Th17 cells in the intestine. Depletion of Th17 cells by crossing *Tak1^flox/flox^;Lyz2-Cre^+/+^* mice with either *Rag1^-/-^* or *Rorc^-/-^* mice abolishes the protection against colitis and CRC (38).

In summary, the genetic alterations of key driver genes in both epithelial cells and immune cells, together with other driver and passenger factors, are crucial in controlling the carcinogenesis and progression of CRC.

## 3 Gut microbiota in CRC development

In healthy people, colonocytes and their metabolism maintain the anaerobic condition and a homeostatic community of commensal bacteria in the gut, which help consume dietary fiber and produce short-chain fatty acids (SCFA) that are beneficial to the host (39). The shift in colonocytes and their metabolism, due to disease, diet, or other damage, will lead to disordered host-commensal symbiosis and dysbiotic microbiota (39). It has been reported that CRC patients have reduced bacterial diversity and richness than healthy people (40, 41). Whereas *Firmicutes*, *Bacteroidetes*, and *Proteobacteria* are the most dominant phyla in the human large bowel (42), *Fusobacterium*, *Peptostreptococcus*, *Porphyromonas*, *Prevotella*, *Parvimonas*, *Bacteroides*, and *Gemella* have been indicated as the most prominent CRC-associated bacteria (10), based on metagenomic sequencing analyses between CRC patients and healthy donors.

Meanwhile, commensal microbiota has been implicated in modulating colitis, CRC, and cancer immunotherapy (43–47). Depending on specific composition, commensal bacteria may exhibit either promoting or suppressing functions in colitis and CRC development (48–52). Long-term antibiotic use in early-to-middle adulthood is associated with an increased risk of colorectal adenoma (53). Similarly, in a mouse colitis model, depletion of microbiota exacerbated tissue damage and shortened survival (54), which is associated with compromised immunity due to the lack of bacterial stimulation. On the other hand, administration of a common antimicrobial additive, Triclosan, alters mouse gut microbiota, increases the severity of colitis symptoms, and promotes colitis-associated CRC in mouse disease models (55). Several CRC-associated bacteria have been identified in cancer patients and animal models (56); however, the understanding of specific bacterial species or strains that induce and modulate anti-tumor immunity is still limited. Below we list the currently identified promoting (**Table 1**) and inhibiting (**Table 2**) microbiota species in colitis and CRC development.

**Table 1:** Identified CRC-promoting microbiota species in preclinical/clinical studies.

Microbiota species	Functions in colitis/CRC	References
Enterotoxigenic *Bacteroides fragilis* (ETBF)	Promotes colitis and CRC	(57–59)
PKS^+^ *Escherichia coli*	Promotes colitis and CRC	(60–62)
*Fusobacterium nucleatum*	Promotes colitis, CRC, and chemoresistance to CRC	(63, 64)
*Campylobacter jejuni*	Promotes colitis and CRC	(65, 66)
*Enterococcus faecalis*	Promotes colitis and CRC	(67, 68)
*Streptococcus bovis*	Promotes colitis and CRC	(69, 70)
*Peptostreptococcus anaerobius*	Promotes CRC initiation and progression	(71, 72)
*Helicobacter pylori*	Induces gastric cancers and positively associates with CRC	(73, 74)
*Mycobacterium avium*	Induces IBD and positively associates with CRC	(75, 76)
*Bilophila wadsworthia*	Positively associates with CRC and causes inflammation	(77, 78)

**Table 2:** Identified CRC-inhibiting microbiota species in preclinical/clinical studies.

Microbiota species	Functions in colitis/CRC	References
*Akkermansia muciniphila*	Inhibits DSS colitis and CRC	(79–81)
*Clostridium butyricum*	Inhibits colitis and CRC	(82–84)
*Odoribacter splanchnicus*	Inhibits colitis and CRC	(38, 85, 86)
Nontoxigenic *Bacteroides fragilis* (NTBF)	Inhibits colitis and CRC	(87)
*Bacteroides* sp. *4_1_36*	Inhibits DSS colitis and negatively associates with CRC	(38)
*Bacteroides* sp. *D20*	Inhibits DSS colitis and negatively associates with CRC	(38)
*Bacteroides uniformis*	Inhibits DSS colitis and negatively associates with CRC	(38)
*Faecalibacterium prausnitzii*	Inhibits TNBS colitis and negatively associates with CRC	(88)
*Holdemanella biformis and Faecalibaculum rodentium*	Inhibit colitis and CRC	(89, 90)
*Clostridium immunis*	Inhibits DSS colitis	(11)
*Peptostreptococcus russellii*	Inhibits DSS colitis	(12)
*Propionibacterium freudenreichii*	Inhibits DSS colitis and induces *in vitro* apoptosis of CRC cells	(91, 92)
*Bifidobacterium bifidum*	Inhibits CRC	(93)
*Lactobacillus coryniformis*	Inhibits CRC	(94)
*Pediococcus pentosaceus*	Inhibits CRC	(95)
*Lactobacillus gasseri*	Inhibits CRC	(96)

### 3.1 Cancer-promoting microbiota species

#### 3.1.1 *Fusobacterium nucleatum*

*Fusobacterium nucleatum* is an anaerobic oral commensal. As a pro-inflammatory species associated with human colitis (97), it has been widely reported to be positively associated with human CRC (98, 99). Accompanied by bacterial dysbiosis in the gut, an infection with this bacteria is prevalent in human colorectal carcinoma (98). In different clinical reports, *Fusobacterium nucleatum* has 8.6% and 13% of colonization in CRC tissues, and is associated with increased microsatellite instability (MSI) and impaired immune responses (100, 101). Furthermore, *Fusobacterium nucleatum* is implicated in accelerating CRC in both human patients and animal models, and is found within metastatic CRC cells in patient biopsies (63, 102). In specific, it adheres to, invades, and induces E-cadherin/β-catenin signaling-mediated oncogenic and inflammatory responses to stimulate CRC carcinogenesis (103–105). CRC cell-resident *Fusobacterium nucleatum* also promotes the secretion of pro-inflammatory cytokines IL-8 and CXCL1, which in turn stimulate the migration and invasion of both infected and noninfected tumor cells (106). In addition, *Fusobacterium nucleatum* promotes chemotherapy resistance of CRC through TLR4- and MYD88-mediated innate immune signaling and autophagy pathway (64).

#### 3.1.2 *Escherichia coli*

*Escherichia coli* is a Gram-negative facultative anaerobic bacterium in the Enterobacteriaceae family, and the adherent-invasive *Escherichia coli* species have been associated with human inflammatory bowel disease (IBD) and CRC (42, 107, 108). Under host inflammatory conditions, the mono-colonization of *Escherichia coli* promotes colitis in *Il10^−/−^* mice and invasive carcinoma in azoxymethane (AOM)-treated *Il10^−/−^* mice (60, 61). Mechanistically, *Escherichia coli* produces the genotoxin colibactin through the non-ribosomal peptide synthetase (NRPS)-polyketide synthase (PKS) hybrid gene cluster (109). Colibactin further alkylates DNA and induces double-strand breaks, aneuploidy, and improper division of colonic epithelial cells (60, 109). In this case, the PKS^+^ *Escherichia coli* enhances tumorigenesis in preclinical CRC models and is enriched in human CRC tissues. Using optical imaging tools, the massive infiltration of inflammatory cells is also observed in PKS^+^ *Escherichia coli*-infected colon tumors, compared with the uninfected group (110).

#### 3.1.3 Enterotoxigenic *Bacteroides fragilis*

The anaerobic Gram-negative Enterotoxigenic *Bacteroides fragilis* (ETBF) is a long-studied human GI pathogen that causes diarrhea and GI inflammation (111, 112). In preclinical models, ETBF potentiates colorectal carcinogenesis in *Apc^Min/+^* mice through STAT3 activation and Th17 cell-dependent colitis (57). Furthermore, ETBF and *Escherichia coli* are detected in biofilms coating human CRCs and precancerous colonic adenomas (58). Tumor-prone mice co-colonized with *Escherichia coli* and ETBF show increased IL-17A level in the colon and DNA damage in colonic epithelia, with faster tumor onset and greater mortality, compared to mice with either bacterial strain alone (58). Similar to *Escherichia coli*, ETBF could produce a metalloprotease toxin BFT (*Bacteroides fragilis* enterotoxin), which has the proteolytic activity to damage the intestinal mucosa and induces a pro-carcinogenic signaling cascade to trigger myeloid-cell-dependent colon tumorigenesis (59, 113).

#### 3.1.4 *Campylobacter jejuni*

*Campylobacter jejuni* is a Gram-negative microaerophilic bacterium that has been considered one of the most widespread infectious diseases in developed countries (114). It produces a genotoxin, cytolethal distending toxin (CDT), which has DNAse activity and leads to DNA double-strand breaks (115). The infection of this species is associated with IBD development in human patients (116), and induces colitis in mouse models (65). In germ-free *Apc^Min/+^* mice, *Campylobacter jejuni* infection promotes colorectal tumorigenesis through the action of CDT (66). As a result, CDT mutation and rapamycin treatment could similarly diminish the tumorigenic capability of *Campylobacter jejuni* (66).

#### 3.1.5 *Enterococcus faecalis*

*Enterococcus faecalis* is a Gram-positive facultative anaerobic bacterium that naturally inhabits the human gastrointestinal tract, and the spread of this bacterium to other organs or tissues can cause severe infection (117). Unlike most other bacteria, this species produces reactive oxygen species (ROS), such as extracellular superoxide (118), thus leading to DNA damage, chromosomal instability, generation of aneuploidy or tetraploidy, and eventually transformation and tumorigenesis of colonic epithelial cells (119, 120). More than half of patients with *Enterococcus faecalis* infective endocarditis (EFIE) of an unidentifiable source are found to have CRC (121). In *Il10^−/−^* mice, *Enterococcus faecalis* promotes colitis development and colorectal tumorigenesis (67, 68).

#### 3.1.6 *Streptococcus bovis*

*Streptococcus bovis* (also known as *Streptococcus gallolyticus*) is a facultative anaerobic Gram-positive bacterium that serves as a causative agent of septicemia and infective endocarditis (IE) in elderly and immunocompromised people (122). Clinical studies have demonstrated a strong association between invasive infections of *Streptococcus bovis* and colon neoplasia (123, 124). Consistently, both *in vivo* and *in vitro* studies validate the pro-inflammatory and CRC-promoting functions of multiple *Streptococcus bovis* strains (69, 70, 125, 126). Since *Streptococcus bovis* is still a normal intestinal tract inhabitant, it may have both passenger and driver functions in CRC tumorigenesis.

#### 3.1.7 *Peptostreptococcus anaerobius*

*Peptostreptococcus anaerobius* is an anaerobic Gram-positive bacterium selectively enriched in fecal and mucosal microbiota of CRC patients (71). Although it can produce tryptophan metabolite indoleacrylic acid, which may attenuate inflammatory response and improve barrier function (12), the direct transfer of *Peptostreptococcus anaerobius* into AOM-treated mice significantly increases colon dysplasia (71). Similarly, in *Apc^Min/+^* mice, it promotes spontaneous CRC development (72). Mechanistically, *Peptostreptococcus anaerobius* selectively adheres to CRC cells, rather than normal colonic epithelial cells, through a surface protein PCWBR2 (putative cell wall binding repeat 2). PCWBR2 stimulates CRC cells to produce pro-inflammatory cytokines, which in turn mediate the local expansion of tumor-supportive MDSCs, TAMs, and TANs (72).

#### 3.1.8 Other potential cancer-promoting microbiota species

Besides the microbiota species that have been validated in either preclinical or clinical functional studies, several other species also show strong correlations with colitis and CRC development and may serve as potential targets for future investigation on CRC management.

*Helicobacter pylori* is a Gram-negative capnophile that can grow in both microaerobic and aerobic conditions. It selectively colonizes the gastric epithelia, and is considered one of the most prevalent bacterial pathogens in humans. *Helicobacter pylori* induces chronic gastritis and is associated with more than 90% of gastric cancers (GC) cases (73, 127, 128), making it a class I carcinogen for GC. Although many reports show that chronic infection of *Helicobacter pylori* is associated with a moderately increased risk of CRC (74, 129, 130), direct evidence from functional studies is lacking.

*Mycobacterium avium* is a microaerobic Gram-positive mycobacterium that is commonly grouped with *Mycobacterium intracellulare* during infection, collectively referred to as *Mycobacterium avium* complex (MAC). *Mycobacterium avium* subspecies *paratuberculosis* (MAP) has long been proposed as a cause of IBD (131, 132); it is increased in IBD patients (75) and can be observed in colon tissues of sporadic CRC patients (76). However, direct functional study of this species is lacking.

*Bilophila wadsworthia* is an anaerobic Gram-negative saccharolytic bacillus that is a major member of sulfidogenic bacteria in human gut (77). It can produce a genotoxin, hydrogen sulfide, which triggers inflammation and hyperproliferation (Yazici et al., 2017). Sulfidogenic bacteria, including *Bilophila wadsworthia*, have a race-dependent association with CRC incidence and is expanded in the population with a higher risk of CRC development (Yazici et al., 2017). In genetically susceptible *Il10^-/-^* mice, diet-induced blooming of *Bilophila wadsworthia* promotes the pro-inflammatory Th1 immune response and an increased incidence of colitis (Devkota et al., 2012). The direct administration of *Bilophila wadsworthia* into specific-pathogen-free (SPF) mice results in systemic inflammation, with reduced body weight and fat mass, apparent hepatosplenomegaly, and elevated serum inflammatory factors (77). However, how *Bilophila wadsworthia* mediated inflammation may impact tumorigenesis is currently unclear.

### 3.2 Cancer-inhibiting microbiota species

#### 3.2.1 *Akkermansia muciniphila*

*Akkermansia muciniphila* is a strictly anaerobic Gram-negative bacterium that resides in the mucus layer and plays a mucin-degrading function in the human intestine (133). It interplays with the intestinal epithelium for nutrition management and controls diet-induced obesity through improved metabolic profiles (134, 135). In preclinical models, *Akkermansia muciniphila* is positively associated with the induction of CRC in mouse recipients of human fecal transplant (136), and is significantly reduced in CRC-resistant *Tak1^flox/flox^;Lyz2-Cre^+/+^* mice (38). In humans, its abundance is decreased in most colitis patients but increased in CRC patients (137). Further studies found that *Akkermansia muciniphila* preferentially expands and colonizes sites of damaged murine mucosa in response to local environmental cues (79), which probably explains the pattern of its distribution and abundance. Notably, *Akkermansia muciniphila* stimulates the proliferation and migration of enterocytes adjacent to the colonic wounds, through FPR1 (formyl peptide receptor 1) and NOX1 (NADPH Oxidase 1)-mediated redox signaling in epithelial cells, thus enhancing the repair of mucosal wounds and protecting mice from chemically induced colitis (79). A similar protective role is observed in another DSS-induced colitis model, with an improved microbial community (80). In AOM/DSS-induced CAC model, *Akkermansia muciniphila* treatment could blunt carcinogenesis by enhancing cytotoxic CD8^+^ T cells (81).

#### 3.2.2 *Clostridium butyricum*

*Clostridium butyricum* is a strictly anaerobic Gram-positive butyrate-producing bacillus that is a dietary probiotic for healthy people and an effective approach to IBD treatment (138). In DSS colitis model, *Clostridium butyricum* directly triggers TLR2/MyD88-dependent IL-10 production by intestinal macrophages in inflamed mucosa to prevent colitis development, and this prevention can be negated in macrophage-specific IL-10-deficient mice (82). In *Apc^Min/+^* mice, *Clostridium butyricum* inhibits intestinal tumor development by decreasing β-catenin expression in Wnt signaling and modulating gut microbiota (83). Similarly, in AOM/DSS model, *Clostridium butyricum* regulates gut microbiota composition and reduces CRC development by inhibiting the NF-κB pathway and promoting apoptosis (84).

#### 3.2.3 *Odoribacter splanchnicus*

*Odoribacter splanchnicus*, a strictly anaerobic Gram-negative bacterium, is a common member of human intestinal microbiota. Although it is enriched in colorectal adenoma and CRC patients (139), recent studies have identified this species as a CRC-inhibiting and -preventive bacterium. In a preclinical model, treatment with wild-mice microbiota renders normal laboratory mice resistant to CRC, and *Odoribacter* ranks among the top increased genera after microbiota reconstruction (13). Strikingly, a recent report has characterized *Odoribacter splanchnicus* as a critical species to protect the host from colitis and CRC (38). This species is highly abundant in a CRC-resistant mouse model (*Tak1^flox/flox^;Lyz2-Cre^+/+^*). Oral transfer of *Odoribacter splanchnicus* into wild-type (WT) mice induces development of immune-suppressive intestinal Th17 cells, and confers resistance against colitis and CRC (38), probably *via* increased productions of IL-17A and IL-22 (140, 141). Similar results are observed in a separate report, in which *Odoribacter splanchnicus* colonization leads to an increase in Foxp3^+^/RORγt^+^ Treg cells, induction of IL-10, and production of SCFA, thus reducing colitis in mouse models (85). Furthermore, treatment of *Odoribacter splanchnicus* supernatant in colon cancer cell lines induces an anti-tumor activity with enhanced apoptosis, and peri-tumoral injection of supernatant significantly decreases CRC formation (86).

#### 3.2.4 Inhibitory *Bacteroides* species

*Bacteroides* species are anaerobic Gram-negative bacilli that are normally mutualistic, making up the most substantial portion of commensal microbiota. Some species, such as ETBF, are reported to promote colitis and CRC, while some others have been identified as anti-tumor players. In both WT and CRC mouse models, treatment of nontoxigenic *Bacteroides fragilis* (NTBF) reduces bacteria-driven chronic colitis and tumor development (87). Recently, *Bacteroides* sp. *4_1_36*, *Bacteroides* sp. *D20*, and *Bacteroides uniformis* are found to accumulate in a CRC-resistant mouse model and significantly inhibit the development of DSS-induced colitis (38). While *Bacteroides* sp. *4_1_36* and *Bacteroides* sp. *D20* are less reported, *Bacteroides uniformis* has a reduced abundance in CRC patients (142) and is reported to improve immunological dysfunction and enhance the gut barrier through the production of butyrate and gamma-aminobutyric acid (143, 144).

#### 3.2.5 *Faecalibacterium prausnitzii*

*Faecalibacterium prausnitzii*, a Gram-positive anaerobic bacterium, is one of the most abundant and important commensal bacteria in human intestine (145). As a key butyrate producer, the abundance of this bacterium is negatively associated with colon tumorigenesis in multiple scenarios (146–148). In IBD patients, the reduction of *Faecalibacterium prausnitzii* is associated with a higher risk of postoperative recurrence (88). Furthermore, in TNBS (2,4,6-trinitrobenzenesulphonic acid)-induced mouse colitis model, both live *Faecalibacterium prausnitzii* and its supernatant exhibit anti-inflammatory effects and markedly ameliorate colitis severity and dysbiosis (88). Mechanistically, metabolites from this species block NF-κB activation in colon epithelial cells and switch the cytokine profile (decreased IFN-γ and IL-12, increased IL-8 and IL-10) (88). However, direct evidence from functional study is in need to determine the role of this species in CRC.

#### 3.2.6 Other potential cancer-inhibiting microbiota species

*Holdemanella biformis* (formerly *Eubacterium biformis*) is a Gram-positive obligately anaerobic bacterium that can release both short chain fatty acids (SCFAs) and long chain fatty acids (LCFAs). The abundance of this species and its family *Erysipelotrichaceae* are reduced in human patients with colon adenomas (89). Although *Holdemanella biformis* is not able to colonize or survive in the mouse intestine, its mouse homologue, *Faecalibaculum rodentium*, exhibits anti-tumorigenic function in both *Apc^Min/+^* and AOM/DSS models (89). Mechanistically, both species produce SCFAs that control protein acetylation and tumor cell proliferation by inhibiting calcineurin and NFATc3 activation (89). *Holdemanella biformis* can also produce 3-hydroxyoctadecaenoic acid (C18-3OH), a LCFA that ameliorates the progression of DSS-induced colitis (90).

*Clostridium immunis*, an anaerobic Gram-positive bacterium, is a relatively new species identified in the *Lachnospiraceae* family (11). *Lachnospiraceae* is dramatically increased in a CRC-resistant mouse model and decreased in Crohn’s disease patients (38, 149), and negatively correlates with CRC development in mouse recipients of human fecal transplant (136). Administration of *Clostridium immunis* protects formerly colitis-prone mice from DSS-induced colitis (11). Further functional and clinical studies are needed to evaluate the potential of this species as a candidate to control CRC development.

*Peptostreptococcus russellii* is a Gram-positive anaerobic bacterium that naturally exists in healthy people. It has an enhanced growth rate in the presence of mucin and is thus identified as a “mucin utilizer” (12), which predicts the potential of being a health-associated commensal, such as the CRC-inhibiting *Akkermansia muciniphila* (133). Oral gavage of *Peptostreptococcus russellii* protects mice from DSS-induced colitis, with significantly ameliorated body mass and histopathological score (12). It also promotes goblet cell differentiation in colon and the expression of goblet cell-specific secreted protein MUC2. Mechanistically, *Peptostreptococcus russellii* encodes the phenyllactate gene cluster and produces tryptophan metabolite indoleacrylic acid, which promotes intestinal epithelial barrier function and mitigates inflammatory responses (12).

*Propionibacterium freudenreichii* is a Gram-positive aerotolerant anaerobe that selectively stimulates the growth of probiotic *Bifidobacteria* through its component DHNA (1.4-Dihydroxy-2-naphthoic acid) (91). In DSS colitis model, the treatment of DHNA shows both preventive and therapeutic effects in disease amelioration (91). A further study shows that this species induces intrinsic apoptosis of CRC cells *via* the production of SCFA (propionate and acetate), and thus enhances cytotoxic activity of TRAIL (TNF-Related Apoptosis-Inducing Ligand)-based therapy in CRC (92). Besides, *Propionibacterium freudenreichii* treatment in healthy people decreases the activity of beta-glucosidase (150), a bacterial enzyme that contributes to CRC development by generating carcinogens. However, direct evidence regarding its function in CRC is lacking.

Recently, the functions of some established natural and engineered probiotics have been investigated in AOM/DSS-induced mouse CRC model. *Bifidobacterium bifidum* treatment increases the abundance of CRC-inhibiting microbiota and the production of beneficial metabolites, thus protecting mice from tumorigenesis (93). *Lactobacillus coryniformis* ameliorates CRC by alleviating inflammation, intestinal microenvironment, and intestinal barrier damage (94). *Pediococcus pentosaceus* inhibits tumor growth in xenograft, and exhibits polyp regression and recovered taxonomic diversity in CRC mice (95). *Lactobacillus gasseri*, accompanied by other prebiotics, reduces the CRC risk *via* the regulation of inflammation, carcinogenesis, and compositional change of gut microbiota (96).. In addition, *Lactobacillus* and *Bifidobacterium* are the most reported probiotics that exert anti-biofilm activity (151). Strikingly, they can form “probiotic biofilms” to fight against other “pathogenic biofilms” (151, 152). Although most *Lactobacillus* strains show an anti-inflammatory effect *in vitro*, only *Lactobacillus fermentum NA4* displays a protective effect *in vivo* (153), suggesting that the beneficial probiotic properties are strain-dependent.

### 3.3 Metabolic products of microbiota on host immunity and CRC

Besides direct interaction, gut microbiota also produces a diverse metabolite repertoire to trigger specific immune responses that may harm or benefit the host indirectly.

#### 3.3.1 Short-chain fatty acids

SCFAs, mainly consisting of acetate, propionate, and butyrate, are a group of organic acids produced by the anaerobic microbial community from carbohydrate fermentation of undigested dietary fiber (154). High fiber diet promotes SCFA production and suppresses CRC development (155), whereas the removal of dietary carbohydrates alters microbiota and results in susceptibility to infectious colitis (156). In general, SCFAs exhibit potential anti-carcinogenic effects in CRC development, with a decreased gut abundance in CRC and adenoma patients (157), consistent with the reduction of butyrate-producing bacteria (158). Loss of FFAR2 (free-fatty acid receptor 2), a SCFA receptor, promotes colon tumorigenesis in mice by reducing gut barrier integrity, over-activating DCs, and promoting CD8^+^ T cell exhaustion (159).

Butyrate and propionate, but not acetate, have a histone deacetylase (HDAC)-inhibiting activity and regulate NF-kB and Wnt signaling in colon epithelial cells. These two SCFAs support basal crypt proliferation in healthy tissues and maintain colonic homeostasis, but inhibit cell growth and induce apoptosis in CRC cell lines (160). Through its receptor GPR109A, butyrate also promotes IL-18 production in intestinal epithelial cells (161, 162), which is a protective cytokine in CRC mouse model (163).

In innate immune cells, butyrate functions intracellularly as a histone deacetylase inhibitor to downregulate IL-6 (164). It inhibits LPS-induced pro-inflammatory mediators in both macrophages and dendritic cells (164, 165). In DSS colitis model, butyrate attenuates the intestinal inflammation by enhancing the M2 macrophage polarization (166). In addition, butyrate and other SCFAs can promote Treg cell generation in mice and ameliorate T cell induced colitis (167, 168).

On the other hand, butyrate may show pro-tumorigenic roles in a context-dependent manner. It inhibits intestinal stem/progenitor proliferation (169), which may suppress advanced cancer but delay tissue damage repair at the early stages of CRC. Butyrate also induces the production of reactive oxygen species (ROS), which may have pro- or anti-tumorigenic functions in different models (170, 171).

#### 3.3.2 Polyamine

Polyamines, such as putrescine, spermidine, spermine, and cadaverine, are aliphatic amines derived from amino acid metabolism in both host tissues and commensal microbiota. They bind to negatively charged macromolecules (DNA, RNA, protein) and regulate a series of cancer-related physiological processes, including cell proliferation, differentiation, apoptosis, angiogenesis, and immune response, etc. (172).

Polyamines are generally considered detrimental metabolites in CRC development. Activated KRAS significantly increases the uptake of polyamines by colon cancer cells (173). Consistently, both polyamines and the key enzyme for polyamine biosynthesis, ornithine decarboxylase (ODC), are dramatically increased in CRC tissues (160), while ODC inhibitor alpha-difluoromethylornithine (DFMO) exhibits promising effects in colon adenoma patients (174). The polyamine catabolic enzyme SMO (spermine oxidase) contributes to ETBF-induced colon tumorigenesis (175), while spermidine directly impacts the colibactin production from PKS^+^ *Escherichia coli* and is required for genotoxic activity (176). Furthermore, SSAT (spermidine/spermine N1-acetyltransferase)-mediated depletion of polyamines inhibits CRC progression and metastasis through the suppression of AKT, GSK3β, and β-catenin signaling (177). Besides, polyamines can regulate T cell activation and macrophage polarization, thus play an important role in CRC microenvironment (160).

CRC patients have an altered microbiota that is closely associated with a higher abundance of polyamines (178). Bacteria biofilm formation in CRC patients is associated with increased cancer cell proliferation and enhanced polyamine metabolism (179, 180), which can be reduced by antibiotic treatment. Furthermore, CRC-associated microbiota not only has an enhanced capacity for converting amino acids into polyamines *via* putrefaction and fermentation pathways (181), but also upregulates polyamine production in host cells (154).

#### 3.3.3 Secondary bile acids

Bile acids (BAs) are synthesized in the liver, stored in the gallbladder, and mostly reabsorbed by ileal epithelial cells during lipid absorption. The small number of unabsorbed BAs are converted into secondary BAs by the microbiota, and become detrimental metabolites to the intestine by contributing to neonatal necrotizing enteritis, IBD, and CRC (182). In the African American population who has a higher risk of CRC, high-fiber low-fat diet suppresses secondary BAs synthesis, resulting in the reduction of CRC biomarkers (183). In CRC mouse model, Apc founding mutation leads to a decreased expression of bile acid apical transporter gene *Slc10A2*, reduced BA reabsorption, and increased secondary BAs, which strongly enhance the gut colonization of CRC-promoting *Streptococcus gallolyticus* (184).

Metaproteomic analysis in stools from CRC patients identifies a heightened oxidative metabolic microenvironment with increased concentrations of DNA-damaging BAs, especially deoxycholic acid (DCA) (185). DCA inhibits gut epithelial cell proliferation *via* the activation of BA receptor FXR (farnesoid X receptor), resulting in the inhibition of wound healing and impaired gut barrier function (186). It also activates the beta-catenin signaling pathway and increases proliferation and invasiveness of CRC cells (187). In *Apc^Min/+^* mice, DCA treatment promotes tumorigenesis with a disrupted intestinal mucosal barrier, activated NLRP3 inflammasome, and increased production of inflammatory cytokines (188). Lithocholic acid (LCA), another typical BA, promotes proliferation and invasiveness of CRC cells (189, 190). Both DCA and LCA are reported to induce cancer stemness in colonic epithelial cells (191).

#### 3.3.4 Other cancer-regulating metabolites

Microbial metabolites from healthy colons are reported to inhibit colon tumorigenesis (192). In specific, *Lactobacillus reuteri* and its metabolite reuterin, which are reduced in mouse and human CRC, could decrease tumor growth and prolong mouse survival by inducing protein oxidation and inhibiting ribosomal biogenesis (192).

Malic acid is a speculated anti-tumor agent produced by *Odoribacter splanchnicus*, based on the gas chromatography-mass spectrometry (GC/MS) analysis of the bacteria supernatant, which induces the apoptosis of colon cancer cells (86).

Hydrogen Sulfide (H_2_S), a toxic gas that can be physiologically produced in the large intestine by commensal microbiota, shows both beneficial and deleterious effects on the intestinal mucosa in a dose- and context-dependent manner (193). In particular, H_2_S ranges from 0.2 mM to 2.0 mM in the mammalian large intestine content and fecal materials, the latter representing the approximate concentration as in the rectum (193). This concentration is critical for the growth of some beneficial microbes, such as *Lactobacillus* (194).

Beneficial AhR ligands: The aryl hydrocarbon receptor (AhR), a ligand-dependent transcription factor with diverse functions in inflammation, detoxification, and homeostasis (195), has been identified as a tumor suppressor in mouse CRC models (196, 197). Ligand-activation of AhR is required for the maintenance of intestinal immune homeostasis and control of inflammation (198, 199). Several microbial tryptophan catabolites, such as indole-3-acetic acid (IAA) and indolepropionic acid (IPA), are natural AhR ligands that can influence the intestinal epithelial barrier (200). Furthermore, indole treatment leads to the repression of inflammation in CRC cell lines, human duodenum-derived organoids, and mouse models (201).

**Figure 1:**
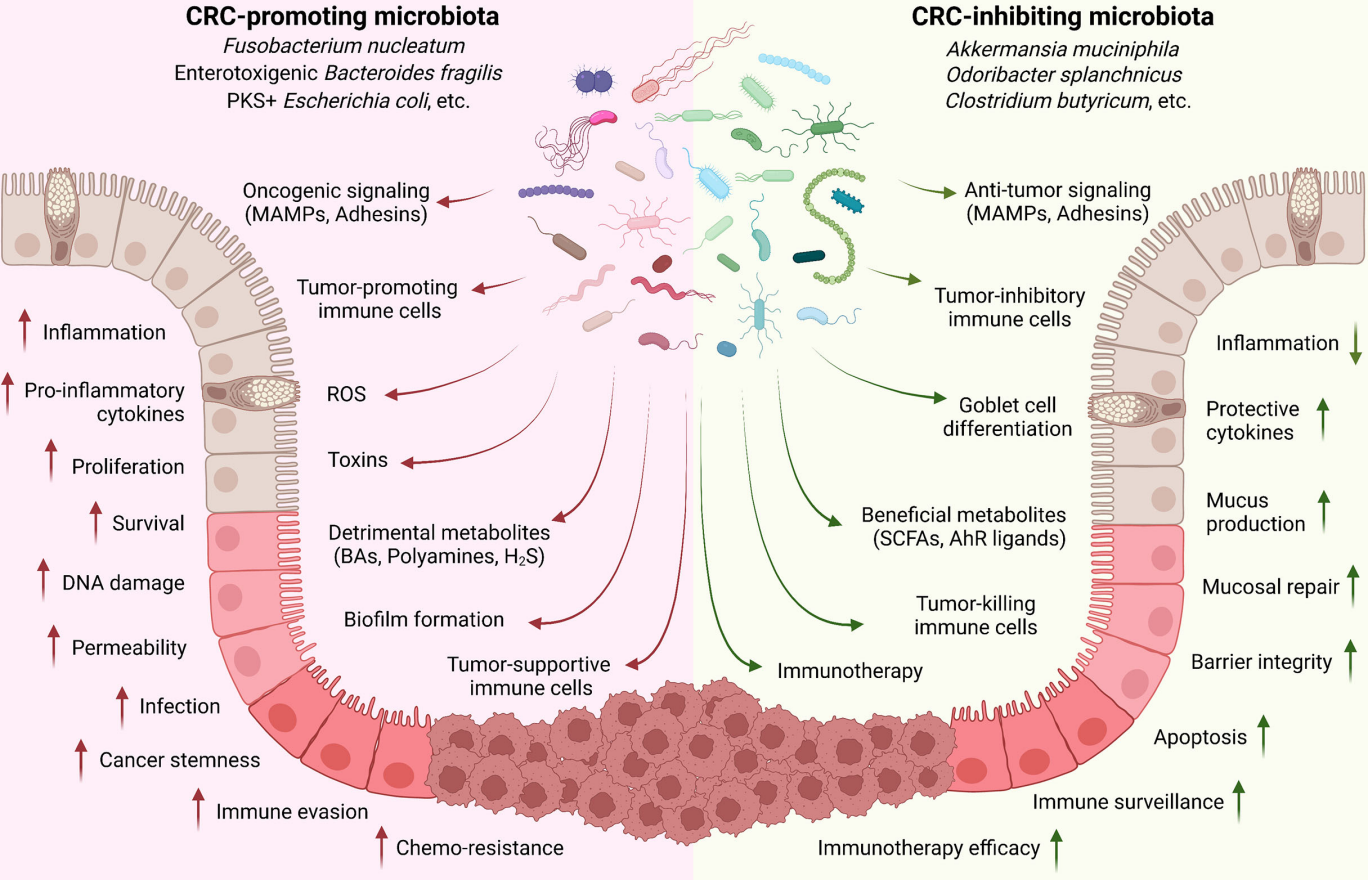
Implication of gut microbiota in CRC development. Commensal microbiota plays critical roles in controlling CRC development. Cancer-promoting microbiota directly adheres to the epithelial cells through MAMPs and adhesins for oncogenic signaling activation (such as Wnt/β-catenin); produce toxins and detrimental metabolites (such as secondary BAs, polyamines, and H_2_S); and induce tumor-associated immune cell populations (such as TAM, TAN, MDSC, and Treg) to regulate the inflammation, tissue damage, cell proliferation and survival, immune evasion, and drug resistance. On the contrary, CRC-inhibiting microbiota can directly trigger the anti-tumor signaling activation in epithelial cells; produce beneficial metabolites (such as SCFAs and AhR ligands); and stimulate tumor-preventing and -killing immune cells (such as CD8, Th1, Th17, and ILC3).

In summary, CRC-regulating microbiota species mainly function through: (1) directly adhering to epithelial cells for oncogenic or anti-tumor signaling activation; (2) producing detrimental (such as toxins) or beneficial (such as SCFAs) metabolites; and (3) inducing tumor-associated or -inhibiting immune cell populations (**Figure 1**).

## 4 Interplay of microbiota and host immune system in regulating CRC development

As key components of the tumor microenvironment, various immune cell populations, particularly tumor-infiltrating immune cells, play critical roles in mediating promotion or inhibition of CRC development. Even in patients after radiation therapy, repopulation of tumor-infiltrating immune cells could be observed after the initial depletion (202). Microbiota communicates with the immune system through various mechanisms, such as Toll-like receptor (TLR) signaling and inflammasome sensing, and regulates inflammation and cancer development through nuclear factor kappa B (NF-κB), type I interferon, and inflammasome pathways (51, 203–205). Local immune system interacts with gut microbiota to control immune responses, tissue damage, and cancer development (11–14).

### 4.1 Innate immune system

Innate immune system is the first line of host defense against pathogens that provides microorganism recognition and plays a critical role in mediating inflammation and cancer development under the stimulation of specific microbiota components (203).

#### 4.1.1 Innate immune signaling

Toll-like receptors (TLRs) are well-defined pattern recognition receptors responsible for pathogen recognition and induction of innate immune responses (204). The densely populated microbiota in the intestinal tract could generate various molecules that can be recognized by TLRs, which leads to NF-κB signaling activation and transcription of multiple cytokines (206). Multiple microbial taxa, particularly some pathogenic bacteria, have been reported to activate TLRs, including PSA-producing *Bacteroides fragilis*, flagellin-producing SFB, *Yersinia enterocolitica*, *Salmonella enterica*, *Helicobacter hepaticus*, *Citrobacter rodentium*, and LPS-producing *Serratia marcescens* and *Escherichia coli* (206, 207). In steady-state, constant recognition of microbiota by TLR4 and TLR1/2 could lead to IL-6, IL-10, and TGF-β production, which is critical for the integrity of intestinal epithelial cells barrier by promoting the expression of tight junction proteins (ZO-1, claudin-1, occludin) and maintaining their proliferation (206). However, aberrant TLR signaling activation in immune cells beneath the IECs could lead to the release of pro-inflammatory cytokines, resulting in acute or chronic intestinal inflammation (204). Therefore, stringent and precise regulation of TLR signaling pathways is essential to maintaining immune balance in the host (204). Particularly, several negative regulators, such as NLRX1, NLRC5, NLRP11, and LRRC25 (208–212), have been identified to control TLR-induced NF-κB signaling pathways at multiple levels, which might be critical for maintaining the delicate balance between bacterial composition, the mucosal immune system, and the intact epithelial barrier.

DNA and RNA sensors: Microbial antigens and potential pathogens are sensed by the host germline-encoded pattern recognition receptors (PRRs) that recognize specific pathogen-associated molecular patterns (PAMPs). As the genetic material, microbial nucleic acids have been identified as the major target for innate immune recognition (213). PRRs that sense intracellular pathogen-derived nucleic acids could mainly divide into three sets, including endosomally localized transmembrane TLRs that sense microbial DNA and RNA in the endolysosomes (214), retinoic acid-inducible gene I (RIG-I)-like receptors (RLRs) that detect pathogen-derived RNA in the cytosol, and cyclic GMP-AMP (cGAMP) synthase (cGAS) and absent in melanoma 2 (AIM2) (215). Type-I interferon (IFN-I) signaling is a major immune signaling initiated by the nucleic acid sensors upon detecting invading pathogens. Double-stranded RNA of one major commensal species, lactic acid bacteria (LAB) is shown to trigger TLR3-mediated interferon-β production by DCs in the gut, which is beneficial in the protection from infection and colitis (216). Besides, LAB could also induce the production of IFN-I through cGAS and RLRs (215). The initiation of IFN-I system by the gut microbiota is shown to be mediated by tonic activation of the cGAS-STING signaling, which is also crucial for innate resistance to DNA and RNA viruses. Further study suggests that activation of cGAS-STING signaling is triggered by membrane vesicle-mediated dispatch of bacterial DNA (217), and is required to link DNA sensing to immune responses (218). Meanwhile, IFN-I signaling may also show a detrimental role in the course of infection with intracellular bacteria. Deficiency of IFN-I signaling through either the genetic deficiency of IFN or IFNAR results increases resistance to oral infection of *Salmonella typhimurium* (219, 220). Therefore, activation of IFN-I signaling mediated by DNA and RNA sensors must be under tight control to maintain a steady intestinal mucosal state in response to pathogen infection and colitis. In the past decade, multiple important regulators have been identified in controlling the IFN-I signaling, such as NLRC5, USP3, USP38, TRIM14, and LRRC25 (209, 212, 221–224). These negative regulators may have potential benefits in maintaining the homeostasis of the gut mucosal system during infections, thus regulating colitis and CRC development.

#### 4.1.2 Innate immune cells

Neutrophils: Regulated by TGF-β and IFN-β signaling, neutrophils may have both tumor-suppressive and -supportive functions (225). Besides the canonical role in mediating the cell phagocytosis and enhancing cytotoxicity, the tumor-associated neutrophils (TANs) can also secrete immunoregulatory and angiogenic factors (225). In CRC, neutrophils are increased with tumor progression (226), and suppress the activity of tumor-infiltrating T cells through the activation of TGFβ (227). Although conflicting results are observed regarding the correlation between neutrophils and survival of CRC patients (228, 229), the high ratio of neutrophils to CD8 T cells is associated with a poor prognosis (230, 231).

Macrophages: In specific conditions, macrophages may differentiate into two distinct types: pro-inflammatory M1 and anti-inflammatory M2 macrophages (232). While total macrophages are increased in CRC with tumor progression (226), M2-type TAMs (tumor-associated macrophages) could promote tumor invasion and angiogenesis, and impair the anti-tumor capacity of T cells (233, 234). Although some studies find that high levels of macrophages are associated with improved prognosis in CRC patients (235, 236), the correlation is opposite in metastatic CRC (237), particularly in elderly patients (202).

MDSCs (myeloid-derived suppressor cells): Characterized by the ability to inhibit both innate and adaptive immune responses, MDSCs are a heterogeneous population of myeloid cells that typically express the common myeloid markers (such as CD33 and CD11b) but lack markers of mature myeloid cells (such as HLA-DR) (238). In both CRC patients and animal models, MDSCs are massively accumulated in the blood, lymph nodes, bone marrow, and tumor sites, particularly in the late stage of cancer (239, 240). An increased MDSC level is correlated with advanced tumor stage and metastasis in CRC patients (241, 242), as well as a shorter survival on chemotherapy (243).

Innate lymphoid cells (ILCs), mainly consisting of natural killer (NK) cells, ILC1, ILC2, and ILC3, are considered the innate counterpart of the T lymphocytes (244). Intestinal ILCs play important roles in controlling epithelial protection, metabolic homeostasis, and development of adaptive immune responses (245–247), and show both pro- and anti-tumor functions in balancing CRC development (248). NK cells have been known for their anti-tumor effects for decades, *via* targeting NCR (natural cytotoxicity receptor) ligands on CRC stem cells and cancer-initiating cells (249). However, developed tumor cells may evade this process by reducing the expression of NCR ligands and upregulating MHC (major histocompatibility complex) class I to suppress NK cell activation (249). Similar to Th1 cells, ILC1s express the transcription factor T-bet and produce IFN-γ and cytotoxic molecules in response to IL-12 and IL-15 for anti-tumor immunity (250). But ILC1-induced inflammation may have negative effects on colitis-associated CRC. ILC2s are rare in the adult human intestine, but are increased in IBD patients (251). Triggered by tumor-derived IL-33, the frequency of IL-13^+^ ILC2s also increases in colorectal tumors (252). Through the production of IL-4, IL-5, and IL-13, ILC2s exhibit context-dependent roles in CRC development (248). ILC3s are frequently accumulated in the intestine and activated by IL-23 for differentiation and production of IL-17A and IL-22 (253). Due to the context-dependent functions of these cytokines (140, 254–263), ILC3s may also present both pro- and anti-tumor functions in CRC development. Notably, the ILCs temper the expansion of bacterial species and protect the gut epithelium in early life (264). After the maturation of adaptive immune system, transient activation of ILC3s by microbial colonization can be extinguished by CD4 T cells (264), indicating that innate and adaptive lymphocytes operate sequentially and in distinct ways during normal development to establish steady-state commensalism and tissue homeostasis. Meanwhile, the loss of ILCs, which express MHC class II for microbial antigen presentation, is associated with dysregulated adaptive immune cell responses against commensal bacteria (246).

The commensal microbiota can regulate the innate immune system through multiple approaches. It may release microorganism-associated molecular patterns (MAMPs), such as flagellin, elongation factor-Tu (EF-Tu), and lipopolysaccharides (LPS), that can be recognized by PRRs on innate immune cells for direct manipulation of functions (265). It may also indirectly influence the innate immune system by metabolites or triggered productions from colon epithelial cells for manipulating the expansion and recruitment of these cells. Several CRC-associated examples are listed.

*Prevotella intermedia*, which is associated with a higher risk of developing CRC (266), evades innate immune control by disabling and killing tissue-infiltrating neutrophils in endodontic infection (267). *Lactobacillus rhamnosus* triggers the anti-inflammatory effects in macrophages and suppresses TNF production through granulocyte-colony stimulating factor (G-CSF)-induced inhibition of c-Jun-N-terminal kinases (JNKs) (268). *Bifidobacterium lactis* attenuates macrophage senescence and induces M2 macrophage polarization (269). Importantly, certain *Lactobacilli* and *Bifidobacteria* species are able to produce butyrate, which in turn control the programming of macrophage for an anti-inflammatory phenotype (270, 271). *Clostridium butyricum* triggers IL-10 production from intestinal macrophages *via* the TLR2/MyD88 signaling pathway and prevents mice from DSS-induced colitis (82). *Fusobacterium nucleatum* administration in mice triggers the increase in tumor-infiltrated immunosuppressive myeloid cells, including MDSCs, TAMs, TANs, and dendritic cells (63). It can also act in a cytokine-independent manner and directly inhibit the cytotoxicity of NK cells against tumors (272). ETBF and *Peptostreptococcus anaerobius* are also reported to trigger the secretion of chemokines that recruit immunosuppressive MDSCs, TAMs, and TANs (59, 72). In addition, *Clostridia* bacteria are reported to modulate the balance of retinoic acid and retinyl esters in intestinal epithelial cells, which further regulates the development of IL-22-producing ILC3s (273, 274).

### 4.2 Adaptive immune system

CD8 T cells are the most potent cytolytic population. Triggered by CRC-derived modulators (such as IL-18) (275), CD8 T cells produce pro-inflammatory cytokines (such as IFN-γ) and cytotoxic molecules for cancer cell clearance (276). Numerous studies have demonstrated the positive association between tumor-infiltrating CD8 T cells with the patients’ prognosis and survival (202, 277). Whereas CD8 cells accumulated at the tumor margin have no effect on survival (278), the ratio of CD8 T cells and Treg cells is a critical determinant of prognosis (279).

CD4 T cells: Different CD4 T helper (Th) cells have distinct roles in regulating CRC development. The cytotoxic Th1 cells are similar to cytolytic CD8 T cells in terms of functions and molecular productions (276), and are positively associated with prolonged survival of CRC patients (280). Treg population plays an immune-suppressive function on multiple immune cell populations through the key cytokines IL-10 and TGF-β (281, 282), but its association with CRC prognosis is controversial (283). Treg infiltration is low in healthy colon, significantly increased in early-stage CRC, but decreased in metastatic cancer (284); and stromal Treg infiltration is 5 times higher than epithelial infiltration in CRC. Further studies find intra-tumoral Treg cells, but not stromal, are associated with increased disease-free survival (285, 286), indicating that the roles of Treg cells may be related to their distribution and immune microenvironment.

Th17 cells are constitutively present in the intestinal lamina propria (LP) due to the activation by microbial flora, such as SFB (segmented filamentous bacteria) (253, 287, 288). Although exhibiting a pro-inflammatory role in autoimmune diseases and host defense against bacteria and fungi (289, 290), Th17 cells in the intestine have an immune-suppressive function (291). In CRC patients, Th17 cells are increased in the tumor and peripheral blood compared with healthy people (292); and the high amount of Th17 cells is associated with tumor progression and a poor prognosis (280, 293). However, the presence of intraepithelial, but not stromal Th17 cells, positively correlates with improved survival (294). The context-dependent function of Th17 population is probably related to the following mechanisms: (1) CRC types. Th17 cells are pathogenic in sporadic CRC models (57, 254–256) but inhibit most CAC models (257–259). (2) Disease stages. Th17 cells may act through altered signaling pathways (such as STAT3) and show distinct roles between the intact epithelial cells in the cancer-initiating stage and the developed tumor cells (295). (3) Differentiation strategies. With the presence of IL-23 or serum amyloid A proteins, Th17 cells could acquire a pathogenic pro-inflammatory phenotype, compared with non-pathogenic Th17 cells induced by IL-6 and TGF-β (296, 297).

The commensal microbiota may regulate the adaptive immune system through multiple approaches. One major aspect is to stimulate and direct the differentiation of several T cell populations in the intestine, particularly Th17 and Treg cells (**Table 3**). Although dispensable for the induction of peripheral Treg cells (313), microbiota tightly controls the development of gut Treg cells. *Bacteroides fragilis* directs Treg development in the gut through its unique immunomodulatory molecule, polysaccharide A (PSA), which mediates the conversion of CD4^+^ T cells into Foxp3^+^ Treg cells that produce IL-10 (302). Further studies illustrate that the antigenic peptides derived from *Akkermansia muciniphila*, *Helicobacter hepaticus*, and several other species induce differentiation of Treg cells in colon and ameliorate intestinal inflammation (303, 304). Similarly, Th17 cell differentiation is mainly directed by specific microbiota strains (49), and some bacteria species have been identified, such as SFB and *Bacteroides fragilis* (57, 87, 287, 314). Recently, *Odoribacter splanchnicus* has been reported to induce the development of immune-suppressive intestinal Th17 cells (38) and Foxp3^+^/RORγt^+^ regulatory T cells (85), both of which limit colitis development in mouse models. Furthermore, microbiota regulates the antigen recognition and tumor-killing function of cytotoxic T cells, such as CD8 and Th1 cells, thus controlling the efficacy of cancer immunotherapy (45, 46, 315, 316).

**Table 3:** Microbiota species in the activation and development of T helper subsets.

Microbiota species	Functions in T helper development	References
Segmented Filamentous Bacteria	Promotes Th17 cells	(287, 288)
*Bacteroides fragilis*	Promotes Th17 cells	(57, 87)
*Odoribacter splanchnicus*	Promotes Th17 cells	(38)
*Citrobacter rodentium*	Promotes Th17 cells	(288, 298)
*Escherichia coli*	Promotes Th17 cells	(288)
*Candida albicans*	Promotes Th17 cells	(299)
*Bifidobacterium adolescentis*	Promotes Th17 cells	(300)
*Bifidobacterium breve*	Promotes Th17 cells	(14)
*Candidatus Arthromitus*	Promotes Th17 cells	(14)
*Staphylococcus epidermidis*	Promotes Th17 cells	(301)
*Bacteroides fragilis*	Promotes Treg cells	(302)
*Helicobacter hepaticus*	Promotes Treg cells	(303)
*Akkermansia muciniphila*	Promotes Treg cells	(304)
*Odoribacter splanchnicus*	Promotes Treg cells	(85)
*Parabacteroides distasonis*	Promotes Treg cells	(14, 305, 306)
*Bifidobacterium infantis*	Promotes Treg cells	(307)
*Bacteroides intestinalis*	Promotes Treg cells	(305)
*Bacteroides caccae*	Promotes Treg cells	(305)
*Bacteroides thetaiotaomicron*	Promotes Treg cells	(305)
*Bacteroides massiliensis*	Promotes Treg cells	(305)
*Bacteroides vulgatus*	Promotes Treg cells	(305)
*Escherichia coli*	Promotes Treg cells	(305)
*Clostridium ramosum*	Promotes Treg cells	(308)
*Fusobacterium nucleatum*	Promotes Treg cells	(308, 309)
*Lactobacillus reuteri*	Promotes Treg cells	(310)
*Lactobacillus murinus*	Promotes Treg cells	(311)
*Faecalibacterium prausnitzii*	Promotes Treg cells	(312)

**Figure 2:**
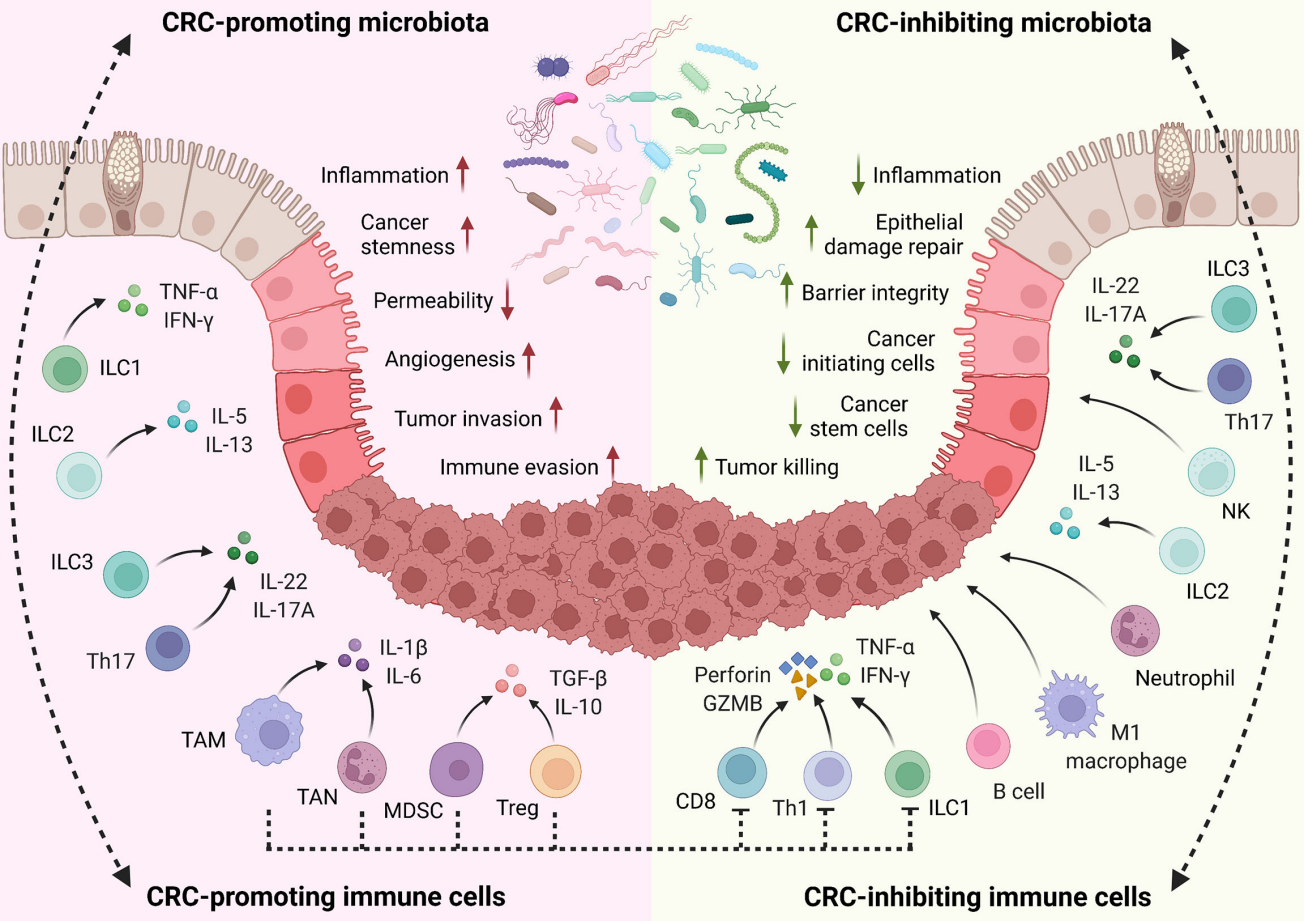
Role of host immune system on CRC. In the host intestine, local immune cells are directly or indirectly stimulated by gut microbiota for activation, proliferation, and differentiation. Meanwhile, the microbiota composition is exquisitely modulated by the immune system. This interplay is important for maintaining homeostasis and plays critical roles in regulating inflammation responses, tissue damage, and CRC development. Through cytokine production and other mechanisms, CRC-promoting immune cells facilitate inflammation, tissue damage, cell proliferation, angiogenesis, tumor invasion, and immune evasion. On the contrary, CRC-inhibiting immune cells enhance epithelial barrier integrity, suppress local inflammation, and eliminate cancer-initiating cells and developed tumors through cytokines, cytotoxic molecules, and other mechanisms. Notably, some immune populations play context-dependent functions in CRC development, based on the disease types, stages, and microenvironment.

In summary, as crucial aspects of the tumor microenvironment, immune cells interplay with the gut microbiota to mediate immune cell functions and control inflammation, anti-tumor immunity, and disease progression (**Figure 2**). Besides the key innate and adaptive immune populations discussed above, functions of other immune cells in CRC development have been previously reviewed in detail (160, 317, 318).

## 5 Gut microbiota in CRC immunotherapy

Microbiota has been identified as a key modulator of cancer immunotherapy (319, 320). Early studies have established the roles of gut microbiota in supporting the CpG-oligonucleotide immunotherapy and the anti-tumor immune responses to cyclophosphamide (CTX) chemotherapy (321, 322). Following research further identified multiple microbiota taxa in enhancing immunomodulatory therapies and controlling tumor-killing efficacy of cytotoxic T cells. *Bifidobacterium* promotes anti-tumor efficacy of anti-PD-L1 therapy by enhancing CD8^+^ T cell priming and accumulation in the tumor microenvironment (45). Similarly, *Bacteroides fragilis* enhances the efficacy of anti-CTLA-4 therapy by triggering a Th1 response and promoting dendritic cell maturation (46). Furthermore, microbiota components from immunotherapy-responding patients lead to improved tumor control, augmented T cell responses, and greater efficacy of immunotherapy in animal models (323, 324). Particularly, *Akkermansia muciniphila* restores the efficacy of PD-1 blockade in non-responders by recruiting CCR9^+^CXCR3^+^CD4^+^ T cells (325). During CTX treatment, the translocation of *Enterococcus hirae* from the intestine to secondary lymphoid organs stimulates IFN-producing CD8^+^ T cells and increases the intratumoral CD8/Treg ratio (315). In addition to the single species, a combination of 11 bacterial strains is reported to improve the anti-tumor efficacy of checkpoint inhibitors with increased tumor antigen-specific CD8^+^/IFN-γ^+^ T cells (316). Microbiota-derived metabolites, such as inosine, could also modulate the response to cancer immunotherapy (326).

Besides traditional and targeted therapies, immunotherapy in CRC treatment has not been widely utilized. Classic vaccination strategies and chimeric antigen receptor (CAR) T cells have shown great clinical benefits, but are accompanied by severe toxicity (327–330). Meanwhile, with the discovery of new druggable immune checkpoints (331), checkpoint blockade therapies have shown good responses in several types of CRC, particularly when combined with chemotherapy (43, 332, 333), but have not been widely investigated. In this case, microbiota-related therapies, particularly with previously identified or even FDA-approved probiotics, may provide novel strategies for CRC treatment. Development of microbiota-related therapies may include: (1) specific elimination of detrimental species by antibiotics or targeted bacteriophage therapy; (2) neutralization of harmful metabolites such as bacterial toxins; (3) supplementary administration of anti-tumorigenic species; (4) fecal transplantation of whole microbiota from healthy donors (particularly for antibiotics-treated patients); (5) diet-driven transition of microbiota or metabolites. While promising results are obtained from increasing clinical trials (334–337), more is needed to validate the efficacy and safety of these strategies. Due to the complexity of microbiota and the numerous effects one species may have on host biology, prudent consideration of any therapeutic approach is necessary based on the host microbiota profile, disease stage, and status of the immune system.

## 6 Gut microbiota and lifestyle risk factors in CRC development

### 6.1 Diet

Dietary factors play important roles in modulating the gut microbiota, which in turn regulates colon inflammation, genotoxic metabolite production, and eventually CRC development (338). On the other hand, gut microbiota composition can influence the physiological effects of dietary components. For example, trimethylamine-N-oxide (TMAO) is a microbiota-dependent metabolite from protein, in particular red meat (339), and an elevated TMAO level is associated with a higher risk of CRC (340). While *Firmicutes* species may contribute to TMAO production (341), *Eubacterium limosum* has the potential to metabolize TMA precursors and reduce TMAO level in gut (342). In addition, dietary carbohydrates, as main fuel sources of the body, have great impacts on the gut microbiota composition and microbiota-related diseases (343). It has been widely reported that the dietary fibers contribute to the reduction of CRC risk (344), mainly through the enhanced production of microbial metabolite SCFAs (345). In this case, specific gut microbiota species, such as *Faecalibacterium prausnitzii*, *Eubacterium rectale*, *Roseburia faecis*, and *Eubacterium halli*, play important roles in the dietary fiber fermentation and SCFA production (346). The abundances of these bacteria are consistently reduced in the gut microbiota of colorectal adenoma patients (347).

### 6.2 Obesity

Lipid metabolism plays an essential role in health management, weight control, and risks to cancers and other infectious diseases (348, 349). Obesity has been reported as an important risk factor of CRC that contributes to approximately 5% of incident cases (348), and gut microbiota is one of the leading factors accompanying and pathogenetically contributing to obesity and its metabolic associates, such as diabetes and cardiovascular diseases (350). The gut microbiota in obese people represents a decreased diversity in phyla and an increased ratio of *Firmicutes : Bacteroidetes* (351), whereas bariatric surgery can reverse these microbial abnormalities (352), associated with changes on dietary habits and macronutrients consumption. In animal models, high-fat diet diminishes the beneficial gut microbiota, such as *Actinobacteria*, *Bifidobacterium*, *Lactobacillus*, and *Akkermansia* (353–356), thus induces gut inflammation, barrier impairment, and an increased risk of CRC development. On the contrary, several well-established probiotics, including *Lactobacillus acidophilus*, *Bifidobacterium lactis*, and *Akkermansia muciniphila*, show an anti-obesity effect (357). The fecal microbiota transplantation from healthy donors, as a promising approach for the treatment of obesity (358), may also be used to control the risk of CRC development.

### 6.3 Alcohol

Alcohol consumption, particularly chronic and moderate to heavy alcohol intake, has been recognized as an important risk factor for CRC, and is closely related to the metastasis and poor prognosis in CRC patients (359). The metabolism of alcohol is actively modulated by the gut microbiota, which regulates ethanol conversion into its metabolites that exert carcinogenic effects in the colon (360). For example, *Enterobacteriaceae Ruminococcus*, and *Bifidobacterium* mediate the production of carcinogenic acetaldehyde from ethanol (361, 362), which accumulates in the colon and greatly contribute to CRC development as ethanol consumption increases. Furthermore, the gut microbiota in alcoholic people is diminished in dominant obligate anaerobes (such as *Bacteroides* and *Bifidobacterium*) and enriched in *Streptococcus* (363), which in turn contribute to CRC development according to their cancer-inhibiting or -promoting functions as discussed in Section 3.

### 6.4 Tobacco

Cigarette smoking has long been identified as a risk factor for CRC development, which is attributed to the synergistic effect of multiple carcinogens, including nicotine, aldehydes, polycyclic aromatic hydrocarbons, heavy metals, volatile organic compounds, and toxic gases (364). The long-term exposure to tobacco smoke induces gut microbial dysbiosis and altered metabolites, and promotes CRC development (365). In specific, cigarette smoke toxicants induce the increases of *Helicobacter*, *Streptococci*, *Firmicutes*, *Peptococcaceae*; as well as the loss of *Bacteroidetes*, *Lachnospiraceae*, and *Lactobacillaceae* (366). Some representative species of these bacterial taxa have been discussed in Section 3 for their CRC-promoting and -inhibiting functions. The increased *Firmicutes : Bacteroidetes* ratio in tobacco users is also associated with obesity, microbial metabolites, and CRC development.

## 7 Conclusions

In the past decade, microbiota has been identified as a critical regulator in maintaining homeostasis, while its imbalance triggers numerous pathological conditions, including CRC. Microbiota may regulate CRC development in multiple approaches: directly by tissue invasion, indirectly by producing metabolites, or by triggering host immune responses. Alteration in the microbiota composition is frequently observed in multiple diseases, while the identification of functional species and strains is limited. In this review, we provide an extensive overview of CRC-regulating microbiota species and how they crosstalk with local enterocytes and the host immune system in controlling disease development, thus offering new insights into our understanding and the development of microbiota-based therapies. Based on their functions, different microbiota species may serve as probiotic supplements or therapeutic targets in the prevention and better treatment of colitis and CRC. The profiling of gut microbiota and metabolites may also serve as novel diagnostic markers to evaluate the CRC risk and prognosis in healthy people and cancer patients.

## Author contributions

R-FW supervised the entire project. CX and R-FW designed and wrote the manuscript. YD, TD, KN, JC, and HYW assisted in specific sections and manuscript editing. All authors contributed to the article and approved the submitted version.

## Funding

This work was in part supported by grants from the NCI, NIH (R01CA101795, R01CA246547, and U54CA210181), Department of Defense (DoD) CDMRP BCRP (BC151081) and LCRP (LC200368) to R-FW.

## Conflict of interest

The authors declare that the research was conducted in the absence of any commercial or financial relationships that could be construed as a potential conflict of interest.

## Publisher’s note

All claims expressed in this article are solely those of the authors and do not necessarily represent those of their affiliated organizations, or those of the publisher, the editors and the reviewers. Any product that may be evaluated in this article, or claim that may be made by its manufacturer, is not guaranteed or endorsed by the publisher.
